# European beech dieback after premature leaf senescence during the 2018 drought in northern Switzerland

**DOI:** 10.1111/plb.13467

**Published:** 2022-10-18

**Authors:** E. R. Frei, M. M. Gossner, Y. Vitasse, V. Queloz, V. Dubach, A. Gessler, C. Ginzler, F. Hagedorn, K. Meusburger, M. Moor, E. Samblás Vives, A. Rigling, I. Uitentuis, G. von Arx, T. Wohlgemuth

**Affiliations:** ^1^ Swiss Federal Institute for Forest, Snow and Landscape Research WSL Birmensdorf Switzerland; ^2^ WSL Institute for Snow and Avalanche Research SLF Davos Dorf Switzerland; ^3^ SwissForestLab Birmensdorf Switzerland; ^4^ Climate Change and Extremes in Alpine Regions Research Centre CERC Davos Dorf Switzerland; ^5^ Department of Environmental Systems Science ETH Zurich Zurich Switzerland; ^6^ Autonomous University of Barcelona (UAB) Cerdanyola del Valles Spain; ^7^ Oeschger Centre for Climate Change Research University of Bern Bern Switzerland

**Keywords:** Bark beetles, bleeding cankers, climatic water balance, crown dieback, *Fagus sylvatica*, tree mortality

## Abstract

- During the particularly severe hot summer drought in 2018, widespread premature leaf senescence was observed in several broadleaved tree species in Central Europe, particularly in European beech (*Fagus sylvatica* L.). For beech, it is yet unknown whether the drought evoked a decline towards tree mortality or whether trees can recover in the longer term.
- In this study, we monitored crown dieback, tree mortality and secondary drought damage symptoms in 963 initially live beech trees that exhibited either premature or normal leaf senescence in 2018 in three regions in northern Switzerland from 2018 to 2021. We related the observed damage to multiple climate‐ and stand‐related parameters.
- Cumulative tree mortality continuously increased up to 7.2% and 1.3% in 2021 for trees with premature and normal leaf senescence in 2018, respectively. Mean crown dieback in surviving trees peaked at 29.2% in 2020 and 8.1% in 2019 for trees with premature and normal leaf senescence, respectively. Thereafter, trees showed first signs of recovery. Crown damage was more pronounced and recovery was slower for trees that showed premature leaf senescence in 2018, for trees growing on drier sites, and for larger trees. The presence of bleeding cankers peaked at 24.6% in 2019 and 10.7% in 2020 for trees with premature and normal leaf senescence, respectively. The presence of bark beetle holes peaked at 22.8% and 14.8% in 2021 for trees with premature and normal leaf senescence, respectively. Both secondary damage symptoms occurred more frequently in trees that had higher proportions of crown dieback and/or showed premature senescence in 2018.
- Our findings demonstrate context‐specific differences in beech mortality and recovery reflecting the importance of regional and local climate and soil conditions. Adapting management to increase forest resilience is gaining importance, given the expected further beech decline on dry sites in northern Switzerland.

## Graphical abstract

Tree and crown mortality in European beech after the 2018 hot drought were more pronounced for trees that showed premature leaf senescence in 2018, for trees growing on drier sites, and for larger trees.

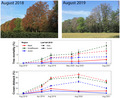

## INTRODUCTION

Severe summer droughts and periods of heat increasingly affect ecosystems globally (Choat *et al*. 2012; Bastos *et al*. 2020; Brodribb *et al*. 2020; Buras *et al*. 2020; Peters *et al*. 2020). Hot or prolonged droughts increase tree mortality rates (Allen *et al*. 2015; Choat *et al*. 2018; Schuldt *et al*. 2020; Senf *et al*. 2020), impact forest growth and carbon sequestration (Ciais *et al*. 2005; Reichstein *et al*. 2013; Cailleret *et al*. 2017) and disrupt mast seeding patterns (Bogdziewicz *et al*. 2020; Nussbaumer *et al*. 2020). Severe droughts can thus act as inciting factors that trigger a spiral of tree decline (Manion 1991), ultimately leading to large forest dieback, changes in community composition and structure, as well as shifts in species distributions (Anderegg *et al*. 2013; Clark *et al*. 2016; Brodribb *et al*. 2020; McDowell *et al*. 2020; Senf *et al*. 2021). These processes will, in turn, affect forest ecosystem services, including timber production, carbon storage, climate and water regulation (Kannenberg *et al*. 2019), as well as compromising sustainable forest management (Suarez & Kitzberger 2008; Bolte *et al*. 2016; Clark *et al*. 2016). From 2018 to 2020, Central Europe experienced an extremely hot drought period (Hanel *et al*. 2018; Boergens *et al*. 2020; Sousa *et al*. 2020) that also severely affected forest ecosystems (Brun *et al*. 2020; Schuldt *et al*. 2020; Senf & Seidl 2021).

As one of the most common deciduous tree species in Central European temperate forests, European beech (*Fagus sylvatica* L.) forms monospecific and mixed stands across broad temperature, moisture and edaphic gradients (Leuschner & Ellenberg 2017). In addition to its ecological value (Winter & Möller 2008; Packham *et al*. 2012), beech is an economically important tree species in many Central European countries, surpassed only by conifers such as Norway spruce and Scots pine (Pretzsch *et al*. 2020). Although beech tolerates a fairly wide range of site conditions (Leuschner *et al*. 2006; Leuschner & Ellenberg 2017), it is known as a moderately drought sensitive species (Gessler *et al*. 2007; Meier & Leuschner 2008; Leuschner & Meier 2018; Leuschner 2020), and was severely affected by the recent period of extreme hot droughts in Europe (Schuldt *et al*. 2020; Walthert *et al*. 2021; Arend *et al*. 2022). Long‐term tree‐ring and forest inventory‐based studies revealed significant growth reductions in response to climatic drought intensity (Bircher *et al*. 2016; Vitasse *et al*. 2019; Pretzsch *et al*. 2020) and increased mortality rates (Archambeau *et al*. 2020). While past drought events mostly affected beech forests at their southern dry distribution limits (Peñuelas & Boada 2003; Allen *et al*. 2010), recent extreme hot droughts have caused extended forest dieback also in the centre of the species' distribution (Leuschner 2020; Schuldt *et al*. 2020). Beech mortality has been related to climate variability (Hember *et al*. 2017; Neumann *et al*. 2017; Archambeau *et al*. 2020) and previous‐year soil moisture anomalies (George *et al*. 2021). However, other factors can predispose beech for crown dieback and mortality, such as shallow soil (Allen *et al*. 2010; Leuschner 2020), high sun‐exposure occurring especially in gaps or at forest edges (Buras *et al*. 2018). Tree size can also influence mortality, but findings are ambiguous: some authors reported that larger (trunk diameter) and taller (tree height) trees are more severely affected by drought because of a higher vulnerability to hydraulic stress, as well as to the higher radiation and evaporative demand experienced by their more exposed crowns (Bennett *et al*. 2015; Rowland *et al*. 2015; Grote *et al*. 2016; Pretzsch *et al*. 2018; Stovall *et al*. 2019; Bottero *et al*. 2021). By contrast, other studies have observed increased vulnerability in shorter and smaller trees, which has been explained by limitations to soil water‐holding capacity in shallow soils and by smaller rooting systems (van Mantgem *et al*. 2009; Giardina *et al*. 2018; Ripullone *et al*. 2020; Nolan *et al*. 2021; Klesse *et al*. 2022). In addition, competition for light, water or nutrients can further aggravate drought stress in beech trees, which might, therefore, be associated with stand density (Gessler *et al*. 2017; Archambeau *et al*. 2020; Castagneri *et al*. 2022).

Premature leaf discoloration and leaf shedding are widely observed in beech in association with summer droughts (Bréda *et al*. 2006; Bigler & Vitasse 2021). On the one hand, leaf shedding may reduce transpiration thus helping trees to avoid embolism by protecting branches, stems and roots from critical water loss (Pollastrini *et al*. 2019; Schuldt *et al*. 2020). On the other hand, premature leaf senescence can also result from hydraulic failure through xylem dysfunction during severe droughts (Wolfe *et al*. 2016; Walthert *et al*. 2021; Arend *et al*. 2022), which is one of several widely reported causes for drought‐induced tree mortality in beech (Leuschner 2020). Other studies suggest that fine root failure more strongly contributes to beech mortality by disrupting the capillary continuum from the soil to the root because of soil water deficit or fine root mortality (Johnson *et al*. 2018; Körner 2019; Martinez‐Vilalta *et al*. 2019). Thermal stress can also increase stomatal conductance and thereby contribute to mortality of leaves, which could expand to twigs and eventually branches (Marchin *et al*. 2022). Hydraulic failure with immediate subsequent tree death occurs only rarely in beech, but embolism can lead to massive crown defoliation and dieback in subsequent years (Brodribb & Cochard 2009; Choat *et al*. 2012; Adams *et al*. 2017; Walthert *et al*. 2021). Although the reasons for drought damage in beech may not be fully understood, crown mortality is a good proxy for vitality, reflecting a reduction in leaf mass and making trees vulnerable to lagged secondary drought impacts (Bréda *et al*. 2006; Schuldt *et al*. 2020). Impaired defence metabolism of drought‐affected beech trees may increase their susceptibility to insect and pathogen attacks. As contributing factors, *sensu* Manion (1991), they potentially accelerate tree death (Anderegg *et al*. 2015a; Brück‐Dyckhoff *et al*. 2019; Huang *et al*. 2020). In addition, bark lesions due to heat and sunburn may also open pathways for pathogen ingress (Butin 2019). As a consequence, such legacy effects can negatively affect tree and forest functioning for several years after a drought event (Kannenberg *et al*. 2019; Kannenberg *et al*. 2020) and contribute to progressive vitality decline. The ability of beech trees to recover depends on the duration and intensity of the drought event, but also on tree‐specific factors such as size and social position (Bennett *et al*. 2015; Anderegg *et al*. 2015b). While recovery from mild droughts can occur within a short time, more severe droughts decelerate recovery processes through structural damage that provokes the need for production of new tissue (Ruehr *et al*. 2019). Regrowth of lost tissue may improve the competitive strength of trees, indicating post‐drought acclimation. Abundant post‐drought re‐growth can also lead to a structural overshoot of aboveground biomass, potentially increasing the trees' vulnerability to recurring droughts (Jump *et al*. 2017; Trugman *et al*. 2018), *i.e*. predisposing them to future decline (Manion 1991).

For a better understanding of drought legacy effects and to disentangle post‐drought acclimation from prolonged impairment or a decline spiral leading to tree death, there is a need to monitor drought‐affected trees, forests and ecosystems over longer periods of time (Gessler *et al*. 2020). Here, we aim to investigate multi‐year drought impacts on beech forests through a unique large‐scale monitoring of 963 beech trees, which showed either premature leaf discoloration during the summer 2018 (824 affected trees) or no visible damage (139 control trees). We conducted the study in two highly drought‐affected regions in northern Switzerland and one less drought‐affected region located further south. We quantified the development of crown dieback and tree mortality as well as secondary drought damage, *i.e*. the presence of bleeding cankers and bark beetle infestations, in these trees for three consecutive years. We also determined the impact of several potential climate‐ and stand‐related (predisposing) factors on mortality and drought legacy processes. We hypothesized that: (i) premature leaf senescence in beech indicates drought damage, eventually leading to partial or full crown mortality, to secondary damage and to continued tree mortality due to drought legacy effects or repeated droughts; (ii) tree mortality and crown dieback, as well as secondary damage, are linked to water deficit, which is related to climatic water balance and soil properties; and (iii) taller trees are more vulnerable to extreme drought.

## MATERIAL AND METHODS

### Study region and tree selection

**Fig. 1:**
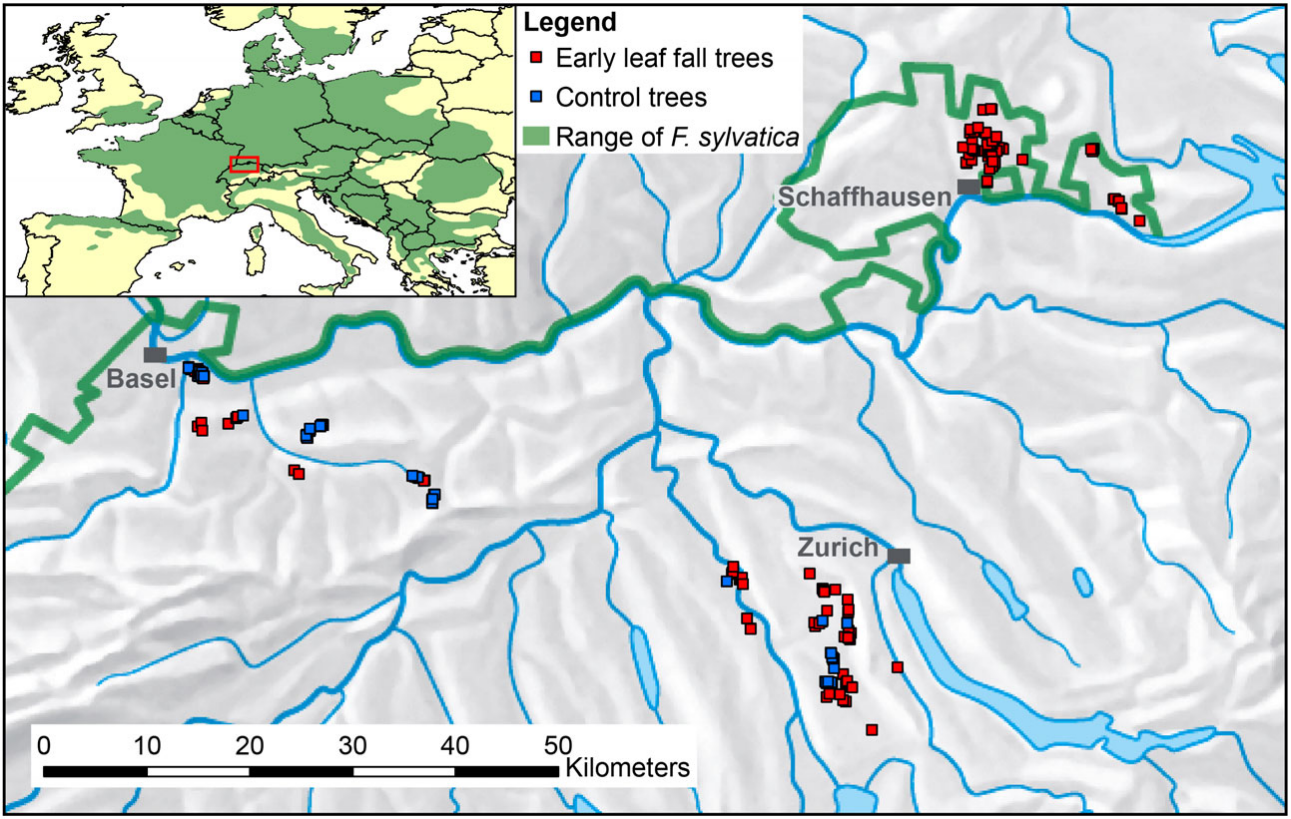
Location of groups of beech trees monitored for drought damage from 2018–2021 in the three study regions near Basel, Schaffhausen and Zurich, in northern Switzerland. Map data derived from © swisstopo, Esri® Data & Maps, and Caudullo *et al*. 2017.

**Fig. 2:**
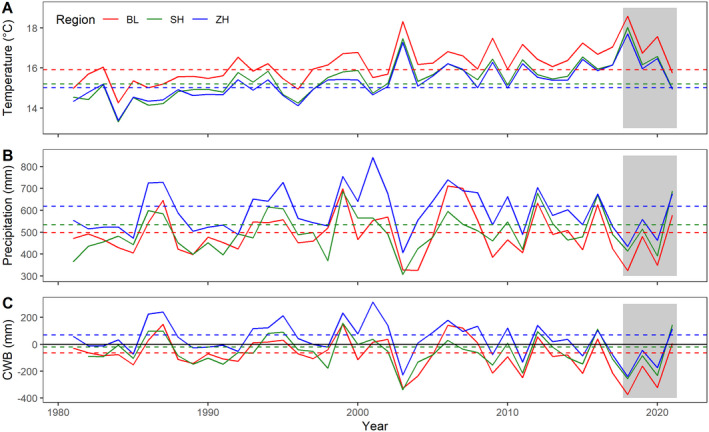
Mean growing season (April to September) air temperature (A), growing season precipitation sum (B), and climatic water balance (CWB), *i.e*. precipitation minus potential evapotranspiration (C) for the period 1981–2021 for the three regions Basel (BL, red line), Schaffhausen (SH, green line) and Zurich (ZH, blue line) in northern Switzerland. Horizontal dashed lines indicate the corresponding value for the climate norm period 1981–2010. and grey shading is the observation period of this study (2018–2021). Climate data derived from © MeteoSwiss (see Table S1 for details).

We conducted our study in three areas in northern Switzerland, where smaller or larger proportions of beech trees showed premature leaf discoloration and leaf fall during the summer of 2018 (Fig. 1). Specifically, we selected an area southeast of Basel (elevation: 271–817 m a.s.l., mean growing season temperature MGT between April and September: 15.9 °C, mean growing season precipitation MGP between April and September: 498 mm) and an area east of Schaffhausen (420–664 m a.s.l., MGT: 15.2 °C, MGP: 534 mm), both severely affected by the drought in summer 2018, as well as a less affected area southwest of Zurich, (360–667 m a.s.l., MGT: 15.0 °C, MGP: 618 mm; Fig. 2, Table S1).

Between the end of August and mid‐September 2018, 963 mature beech trees (395 trees in Basel, 300 trees in Schaffhausen, 268 trees in Zurich) in patches of three to five trees (207 tree groups in total) were selected and permanently marked in pure and mixed, moderately managed beech stands with natural regeneration (Wohlgemuth *et al*. 2020). Study trees were dominant and co‐dominant trees with diameters mostly >30 cm that had no or very few dead branches. The initial set included 824 trees that exhibited premature leaf discoloration of at least 50% of the crown volume or even partial leaf shedding before mid‐September (hereafter referred to as early leaf fall trees), and 139 trees whose leaves were still green by mid‐September (hereafter referred to as control trees). In the area of Schaffhausen, most of the trees were showing signs of premature leaf senescence and, thus, it was not possible to select control trees.

### Crown condition monitoring and individual tree parameters

For three growing seasons, we assessed the crown condition of drought‐damaged and control trees and used multiple regression analysis to study the potential influence of several tree, stand, site and climate parameters as potential predisposing factors on the observed damage and to determine their relative importance. Crown condition of all trees was assessed for the first time at the end of the summer drought 2018 (*i.e*. in August and September 2018). Further crown condition assessments were conducted in spring 2019 (April, May) and in the summers of 2019, 2020 and 2021 (July, August). Specifically, we visually estimated crown dieback as the volume proportion of dead branches (including lost branches) relative to the volume of the total potential crown of the healthy tree, thereby excluding naturally dying branches in the shaded part of the crown (Dobbertin *et al*. 2016). Crown dieback was estimated in 5% classes, ranging from 0% (no crown dieback) to 100% (fully dead crown). A branch (>4 cm) was considered dead if no living tissue (leaves, buds) was present. Recently lost branches (since August 2018) were also included in the proportion of dead branches by estimating their original branch volume. As a proxy of crown defoliation, crown transparency was estimated as the percentage of leaf loss (*i.e*. leaf buds that do not form or that have not sprouted/burst in relation to the maximum possible leaf volume) compared to a reference tree with a fully foliated crown from a photo guide with species‐specific reference standards (Eichhorn *et al*. 2016). Dead branches were excluded from the assessment of crown defoliation. Defoliation was estimated in 5% classes, ranging from 0% (no defoliation) to 100% (fully defoliated crown). As a measure of tree recovery, we assessed the proportion of branches that produced fresh leaf biomass in the crown after the 2018 drought. Study trees that had no remaining visible living leaf tissue were considered as dead (tree mortality). As secondary damage parameters, the presence of bark beetle holes and of fresh bleeding cankers on each trunk was recorded from the root collar to a height of 2 m above ground in summer 2019, 2020 and 2021. All observations in a year were performed by the same expert‐trained team and teams were calibrated against each other.

For each selected tree, we measured its diameter at breast height (DBH) with a measuring tape, assessed its social position (dominant, co‐dominant) as well as the aspect and topography of its environment according to the ICP Forests definition and guidelines of the National Forest Inventory (Düggelin *et al*. 2020). Tree positions were recorded using a GNSS receiver (GeoXH 6000 DGNSS, Trimble Navigation, Sunnyvale, CA, USA). Post‐processed coordinates achieved a horizontal precision of 0.1–2.0 m. As trees are expected to be more vulnerable to drought damage with increasing height and decreasing distance to the forest edge, these parameters were determined based on tree coordinates, a vegetation height model and a forest mask from the Swiss National Forest Inventory (Waser *et al*. 2015). During the initial survey in early autumn 2018, mast seeding of each tree was visually assessed in four classes (0 = absent, 1 = scarce, 2 = medium, 3 = high; Rohmeder 1972), because this may render trees more susceptible to drought (Hacket‐Pain *et al*. 2017).

### Climate and stand parameters

As further potentially predisposing factors, we considered site climate and stand parameters. We extracted temperature, precipitation and incoming solar radiation for the location of each tree group from MeteoSwiss data downscaled to a 25‐m grid (Source: MeteoSwiss). From these parameters, the climatic water balance (CWB) for the location of each tree group was calculated as the difference between precipitation and potential evapotranspiration. The latter was approximated from temperature and solar radiation according to Turc (1961). In the statistical models, we considered the growing season CWB (April to September) averaged over the years 2013 through 2019 because these years were characterized by repeated drought phases, which potentially may have affected tree health.

Furthermore, soil properties were considered as potential predisposing factors. Average soil depth for each tree group was determined with a steel auger that was driven into the ground up to a maximum depth of 120 cm (n = 2 per group). Additionally, a sample of the mineral topsoil (0–10 cm) was collected from the centre of each tree group with a steel hand probe to determine soil pH of a 1:2 soil:0.01 M CaCl_2_ suspension, using a pH meter in the lab. Additional soil properties (gravel volume and clay content) were derived from digital soil maps based on machine learning models (Baltensweiler *et al*. 2021). For both parameters, we calculated the weighted mean of all values up to a depth of 100 cm.

Increased competition is another factor potentially aggravating drought damage. We determined the competition index according to Hegyi (1974) for each study tree to quantify competition among individual trees. For this purpose, we measured the distances between each of our study trees (target trees *i*) and all of their neighbouring trees *j* with a DBH ≥20 cm within a 10‐m radius using a Vertex clinometer (Haglof Vertex 3). DBH of all target trees *i* and neighbouring trees *j* was measured with a measuring tape in summer 2021. Based on these data, we calculated the competition index CI_*i*_ for each tree according to Hegyi (1974):

$${\mathrm{CI}}_{i}=\sum_{j=1}^{n}\frac{{\mathrm{DBH}}_{j}/{\mathrm{DBH}}_{i}}{{\text{Distance}}_{\mathrm{ij}}}$$

### Data analysis

We used binomial generalized linear mixed effects models (GLMMs) with logit link functions to quantify the influence of explanatory variables on the proportion of dead trees per tree group (cumulative tree mortality), crown dieback percentage of individual trees, the presence of bark beetle holes on each trunk, and the presence of fresh bleeding cankers on each trunk. Logged trees were excluded from all models because data for these was incomplete. The initial models contained the explanatory variables ‘Leaf fall 2018’, ‘Region’, the climatic water balance for the growing season (April to September) averaged over the years 2013–2019 (‘CWB’), the time interval between August 2018 and the survey (‘Time interval’), diameter at breast height (‘DBH’), ‘Tree height’, ‘Competition index’, the distance from the nearest forest edge (‘Forest edge distance’), mast seeding status 2018 (‘Seed mast 2018’), ‘Social position’, and the proportion of ‘Crown dieback’ (in the models for bark beetles and bleeding cankers) for each tree, as well as mean ‘Soil depth’, mean ‘Soil pH’, ‘Gravel content’, ‘Clay content’ averaged for each tree group as fixed effects (Table 1, Table S4). A quadratic term for ‘Time interval’ was included in the models for crown dieback and bleeding cankers to account for the non‐linear temporal development of these response variables. To avoid convergence problems due to overfitting, ‘Tree height’ and ‘Gravel content’ had to be excluded from the models for bark beetles and bleeding cankers. These models also included two‐way interactions of ‘Leaf fall 2018’ with some other explanatory variables. Continuous explanatory variables were standardized to zero mean and unit variance using the function ‘decostand’ from the R package ‘vegan’ (Oksanen *et al*. 2019) to make effect sizes comparable. ‘Tree’ nested within ‘Tree group’ nested within ‘Region’ was included as random effect to account for spatial autocorrelation of trees within the same tree group and for temporal autocorrelation among multiple observations of the same tree. Models were fitted using the R package glmmTMB (Brooks *et al*. 2017). Variance inflation factors (VIF) were calculated based on the models containing all fixed and random effects using the R package ‘performance’ (Lüdecke *et al*. 2021) to check for multicollinearity among factors. Factors with VIF >5 were successively removed, starting with the factor with the highest VIF until VIF <5 for all parameters. This resulted in removing ‘Region’ from all models. Homogeneity and homoscedasticity of simulated scaled residuals was confirmed using the R package DHARMa (Hartig 2021). A stepwise model reduction procedure was applied, in which individual interactions and main factors were systematically removed, while respecting the principle of marginality to find the most parsimonious model. In each step, we removed the factor that resulted in the largest reduction of Akaike's information criterion (AIC) in comparison to the previous model. This procedure was repeated as long as a factor removal reduced AIC by >2 (Zuur *et al*. 2009). All analyses were performed with the statistical software R (R Development Core Team 2021).

**Table 1:** Response and explanatory variables used in regression models.

Variable	Description	Type	Survey
Response variables			
Bark beetles	Presence of bark beetle holes on the trunk	binary	*t*_1_, *t*_2,_ *t*_4_, *t*_5_
Bleeding cankers	Presence of fresh bleeding cankers on the trunk	binary	*t*_1_, *t*_2_, *t*_4_, *t*_5_
Crown dieback	Proportion of crown dieback	proportion	*t*_0_, *t*_1_, *t*_2_, *t*_4_, *t*_5_
Tree mortality	Proportion of dead trees per tree group	proportion	*t*_0_ – *t*_5_
Explanatory variables			
Clay content^1^	Weight fraction of clay in the fine earth	proportion	modelled
Competition index^1^	Hegyi's competition index, showing competition by neighbouring trees (*r* ≤ 10 m)	continuous	*t*_5_
Crown dieback^2^	Proportion of crown dieback	proportion	*t*_2_, *t*_4_, *t*_5_
CWB^1,2^	Mean climatic water balance of the growing season for 2013–2019	continuous	modelled
DBH^1^	Tree trunk diameter at breast height	continuous	*t*_0_
Forest edge distance^1^	Distance from the nearest forest edge	continuous	modelled
Gravel content^1^	Volume fraction of gravel in the soil	proportion	modelled
Leaf fall 2018	Timing of leaf fall in 2018 (0 = normal, 1 = early)	binary	*t*_0_
Region	Study region (Basel, Schaffhausen, Zurich)	categorical	–
Seed mast	Mast seeding status 2018 (0 = absent, 1 = scarce, 2 = medium, 3 = high)	ordinal	*t*_0_
Social position	Social position of the tree (1 = dominant, 2 = co‐dominant, 3 = sub‐dominant)	ordinal	*t*_0_
Soil depth^1^	Mean soil depth at stand level	continuous	*t*_4_
Soil pH	Mean soil pH at stand level	continuous	*t*_4_
Time interval^1,2^	Time interval between August 2018 and survey date	continuous	–
Tree height^1^	Tree height derived from the LFI vegetation height model	continuous	modelled

Superscript numbers after explanatory variables indicate two‐way interactions with ‘Leaf fall 2018’ in the model with crown dieback (1), in the model with bleeding cankers (2) and in both models (1,2). Competition index, calculated according to (Hegyi 1974). CWB, climatic water balance of the growing season (April to September) averaged over the years 2013 through 2019, *i.e*. precipitation minus potential evapotranspiration. Type, variable type. Survey, dates of survey (*t*_0_ = August 2018, *t*_1_ = April 2019, *t*_2_ = August 2019 *t*_3_ = May 2020, *t*_4_ = August 2020, *t*_5_ = August 2021). Tree mortality was recorded during all six surveys. Modelled, variables derived from interpolated climate data, digital soil maps and vegetation height models.

## RESULTS

### Tree mortality

**Fig. 3:**
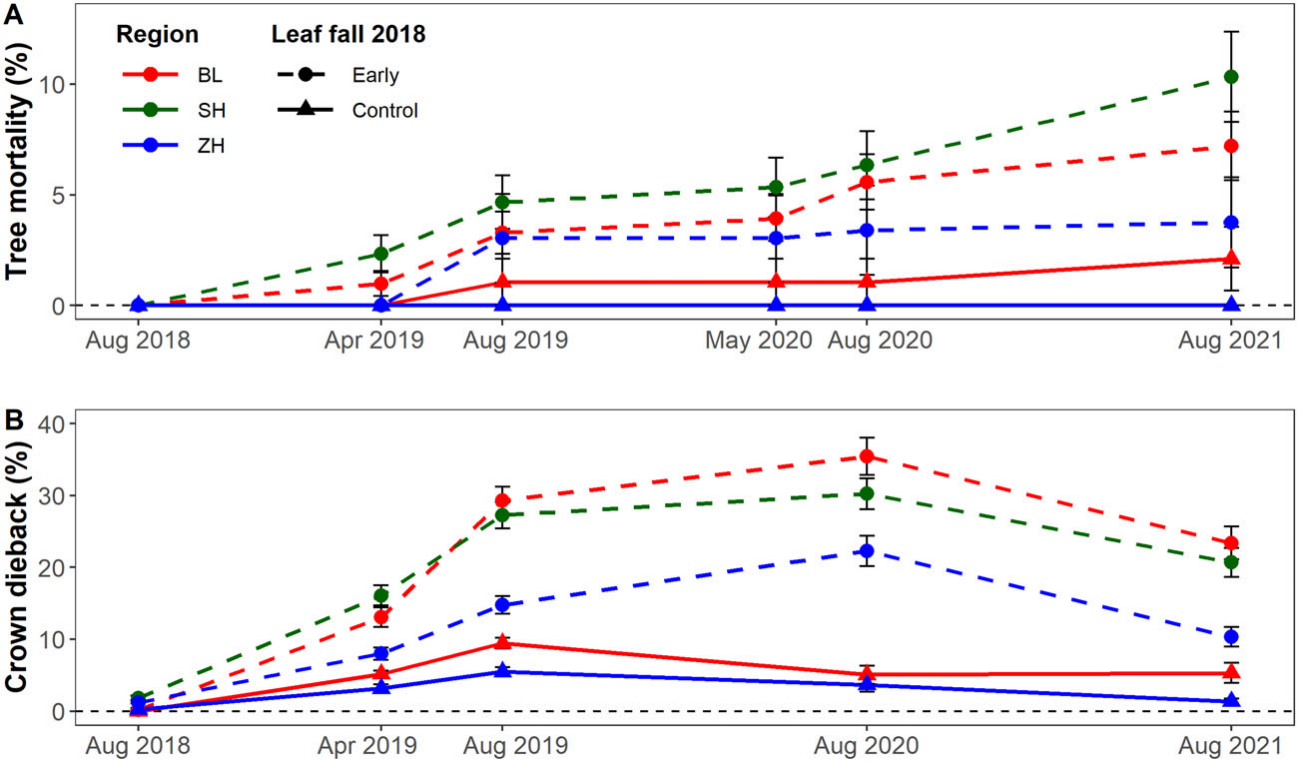
Development of cumulative tree mortality (A) and crown dieback (B) (mean ± SE) in early leaf fall (dashed lines) and control trees (solid lines) in the three regions, Basel (BL), Schaffhausen (SH) and Zurich (ZH) in northern Switzerland, from 2018 to 2021. Logged trees were excluded, and in Panel B trees were excluded if time series were incomplete (N = 745; for numbers see also Table S3).

All study trees were alive at the beginning of the project in August 2018. Thereafter, the proportion of dead trees in all regions continuously increased, with mean annual mortality rates between 2018 and 2021 of 2.1% and 0.5% in early leaf fall and control trees, respectively. In summer 2021, cumulative mortality (excluding logging) reached 7.2 ± 1.1% and 1.3 ± 0.9% (mean ± 1 SE) for early leaf fall and control trees, respectively. In the same year, the cumulative percentage of standing dead trees with respect to the total number of early leaf fall trees was 10.3 ± 2.0% in Schaffhausen (31 of 249 trees), 7.2 ± 1.6% in Basel (22 of 178 trees) and 3.8 ± 2.0% in Zurich (4 of 196 trees), whereas the values for control trees were only 2.1 ± 1.4% in Basel (2 of 81 trees) and 0.0 ± 0.0% in Zurich (0 of 41 trees; no observations in Schaffhausen), respectively (Fig. 3A, Table S2). A substantial number of trees (207, *i.e*. 21.2 ± 2.5%) had been logged by 2021, often to prevent damage to people and infrastructure due to uncontrolled branch and trunk breakage in damaged trees. These logged trees were excluded from the analysis because their crown condition at the time of felling was unknown and time series for these trees were incomplete. Overall, mortality and logging combined accounted for a loss of 25.6% of the 963 study trees by summer 2021. While the health status of logged trees at the time of cutting could not be recorded, only 19.7% of these trees showed ≥80% crown dieback or were dead in the last survey before logging. At this time (the last survey before logging), mean crown dieback of logged trees was 63% higher than the highest observed mean crown dieback for all other trees (41.3 ± 2.4% *versus* 25.3 ± 1.2% for logged and remaining trees, respectively).

Mixed model analysis confirmed that tree mortality was significantly higher in early leaf fall trees (*P* = 0.001) and in locations with a larger CWB deficit (*P* = 0.005; Table 2A). Beech trees with a larger DBH (*P* = 0.04) and trees that were under higher competition (*P* = 0.03) also showed higher mortality. Finally, mortality increased monotonously over time (*P* < 0.001).

**Table 2:** Results of binomial generalized linear mixed effect models after stepwise model reduction for tree mortality (A), proportion of crown dieback (B), presence of bleeding cankers (C), and presence of bark beetle holes (D). All models used a logit link function and included ‘Tree’ nested within ‘Tree group’ and ‘Region’ as random effect. Models were based on data from N = 176 tree groups for tree mortality, from N = 745 individual trees for crown dieback, bleeding cankers and bark beetles. Continuous explanatory variables were standardized to zero mean and unit variance. Significant explanatory variables are **in bold** and non‐significant factors that were dropped during model reduction are indicated with ‘ns‘. Factors that were not included in the initial models are indicated with ‘–’.

Explanatory variable	(A) Tree mortality			(B) Crown dieback			(C) Bleeding cankers			(D) Bark beetles		
	Est.	SE	*P*	Est.	SE	*P*	Est.	SE	*P*	Est.	SE	*P*
Leaf fall 2018	5.17	1.63	**0.001**	1.03	0.27	**<0.001**	1.04	0.34	**0.002**	0.17	0.85	0.839
Competition index	1.62	0.73	**0.026**	0.09	0.08	0.303	ns	ns	ns	−0.09	0.37	0.804
Seed mast 2018	−1.08	0.68	0.109	ns	ns	ns	ns	ns	ns	ns	ns	ns
Social position	0.39	0.78	0.622	ns	ns	ns	ns	ns	ns	ns	ns	ns
DBH	0.10	0.05	**0.035**	0.45	0.19	**0.015**	0.86	0.12	**<0.001**	0.34	0.37	0.350
Tree height	0.10	0.11	0.348	0.18	0.08	**0.019**	–	–	–	–	–	–
Clay content	0.09	0.07	0.189	0.41	0.08	**<0.001**	ns	ns	ns	ns	ns	ns
Time interval	0.08	0.01	**<0.001**	0.33	0.01	**<0.001**	1.08	0.30	**<0.001**	2.93	0.26	**<0.001**
Time interval^2^	–	–	–	−0.01	0.00	**<0.001**	−1.72	0.14	**<0.001**	–	–	–
Gravel content	0.07	0.04	0.109	0.22	0.09	**0.010**	–	–	–	–	–	–
Soil pH	−0.06	0.33	0.851	ns	ns	ns	0.23	0.11	**0.044**	0.18	0.29	0.538
CWB	−0.02	0.01	**0.005**	−0.33	0.09	**<0.001**	−0.61	0.14	**<0.001**	0.48	0.79	0.545
Forest edge distance	−0.01	0.00	0.135	−0.11	0.08	0.170	−0.26	0.12	**0.032**	0.14	0.29	0.631
Soil depth	−0.01	0.01	0.565	0.03	0.08	0.685	0.04	0.11	0.725	0.05	0.27	0.844
Crown dieback	–	–	–	–	–	–	0.39	0.08	**<0.001**	1.50	0.21	**<0.001**
Leaf fall 2018 × DBH	–	–	–	−0.28	0.19	0.148	–	–	–	–	–	–
Leaf fall 2018 × Tree height	–	–	–	0.23	0.19	0.238	–	–	–	–	–	–
Leaf fall 2018 × Time	ns	ns	ns	0.04	0.01	**<0.001**	−0.54	0.31	0.072	ns	ns	ns
Leaf fall 2018 × CWB	–	–	–	ns	ns	ns	ns	ns	ns	−0.57	0.85	0.505

Est., estimate; SE, standard error; *P*, *P*‐value. Time interval^2^, quadratic term for factor time interval. For description of explanatory variables see Table 1. Interaction terms that were dropped during model reduction were not included in this table.

### Crown dieback

In the first survey in early autumn 2018, crown dieback was very low, with an average of 1.2 ± 0.1% dead branches in the crown for early leaf fall trees and 0.1 ± 0.1% for control trees (Fig. 3B, Table S3A). Crown dieback steadily increased and peaked in August 2019 for control trees at 8.1 ± 0.4% and in August 2020 for early leaf fall trees at 29.2 ± 1.3%, before decreasing again in August 2021 to averages of 5.4 ± 1.1% and 25.5 ± 1.4%, respectively. Crown dieback for early leaf fall trees reached peak values of 35.4 ± 2.6% in Basel, 30.2 ± 2.2% in Schaffhausen and 22.3 ± 2.1% in Zurich in 2020. For this analysis, we excluded all logged and dead trees, as data series were not complete. In all surveys, crown dieback was about five times higher in early leaf fall trees than in control trees.

Crown dieback was best explained by climate, soil and tree size variables. It was higher at sites with more negative CWB (*P* < 0.001; Table 2B). Dieback was elevated on soils with a higher gravel content (*P* = 0.01) and with a higher clay content (*P* < 0.001). Larger trees (DBH; *P* = 0.02) and taller trees (tree height; *P* = 0.02) also exhibited elevated dieback. The significant interaction of time interval × leaf fall shows that the increase in crown dieback over time was significantly higher in trees with early leaf fall compared to control trees (*P* < 0.001). Crown dieback peaked in 2020 and was followed by lower values due to partial recovery, as indicated by the significant quadratic term of the time interval between August 2018 and the surveys (*P* < 0.001). All other variables in the initial model did not significantly influence crown dieback.

### Secondary damage

**Fig. 4:**
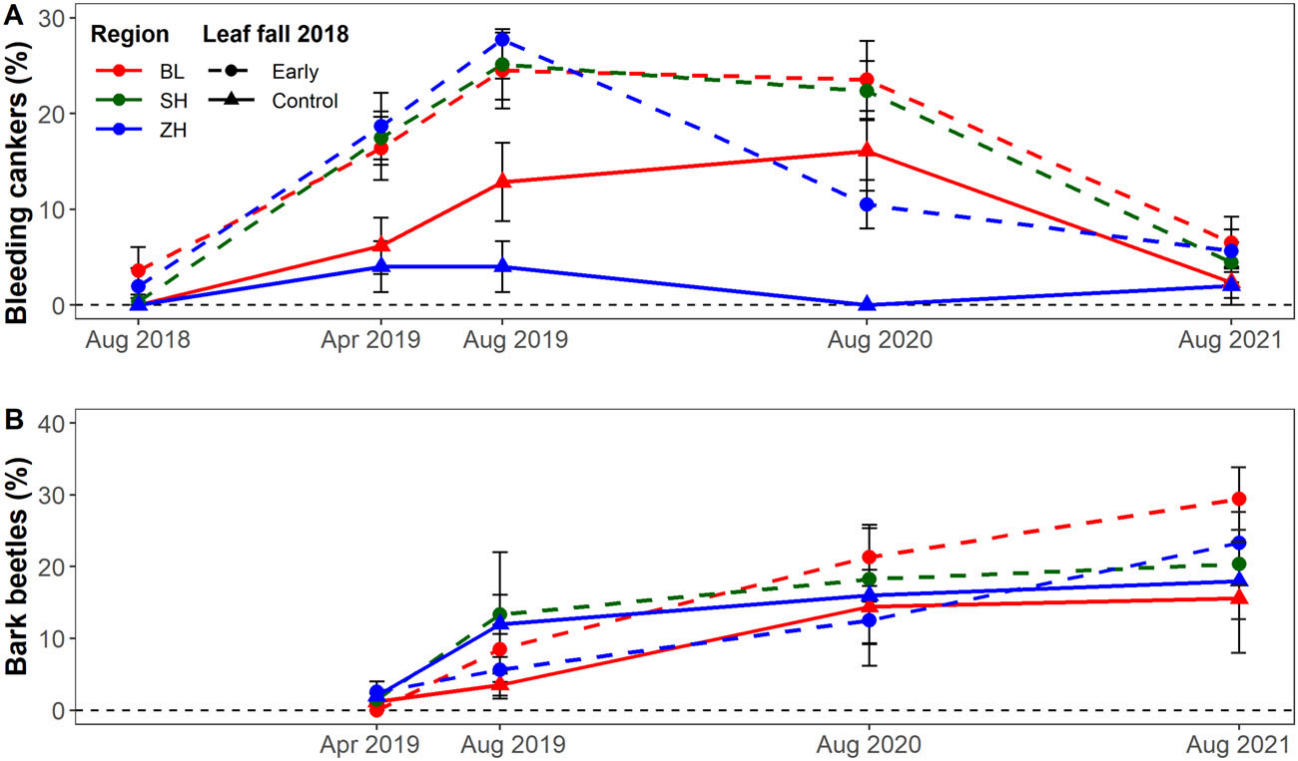
Presence of fresh bleeding cankers (A) and bark beetle holes (B) in early leaf fall (dashed lines) and control trees (solid lines) in the three regions Basel (BL), Schaffhausen (SH) and Zurich (ZH) in northern Switzerland from 2018 to 2021. Trees with incomplete time series due to logging were excluded (N = 745; for numbers see also Table S3).

The proportion of trees with bleeding cankers was <2% in the first inventory in 2018 (Fig. 4A, Table S3B). In the aftermath of the 2018 drought, proportions peaked in summer 2019 for trees in Zurich at 26.5% and 4.9% for early leaf fall and control trees, respectively, and in summer 2020 for trees in Basel (23.2% *versus* 16.0%, respectively) and Schaffhausen (26.3% for early leaf fall trees). Thereafter, the proportion of trees with bleeding cankers decreased to <6% in summer 2021. Proportions were more than three times higher in early leaf fall than in control trees. The cumulative proportion of trees with bark beetle holes continuously increased in the 3 years after the 2018 drought (Fig. 4B, Table S3C). In 2021, bark beetle holes were present on 21.5% of all trees. There was a non‐significant tendency that early leaf fall trees were more affected than control trees (22.8% *versus* 14.8%; *P* = 0.84). The highest percentage of affected trees was found among early leaf fall trees in Schaffhausen (31.5%).

Mixed models showed that bleeding cankers were more frequently found on early leaf fall trees (*P* = 0.002; Table 2C) and on trees with higher crown dieback values (*P* < 0.001). They were most frequently observed 1–2 years after 2018, as indicated by the significant quadratic term for time interval (*P* < 0.001). Presence of bleeding cankers was also more frequent on trees with a larger DBH (*P* < 0.001) and at locations with more negative CWB (*P* < 0.001) and higher soil pH (*P* = 0.05), as well as on trees located closer to the forest edge (*P* = 0.03). The other factors in the initial model did not significantly influence bleeding canker occurrence. The number of trees with presence of bark beetle holes increased with the percentage of crown dieback (*P* < 0.001; Table 2D) and with the time elapsed after August 2018 (*P* < 0.001). None of the other factors in the initial model significantly influenced the presence of bark beetle holes.

## DISCUSSION

The large‐scale monitoring of nearly a thousand beech trees for three consecutive years following the extreme 2018 drought showed continuously increasing tree mortality, which was potentially exacerbated by the repeated hot and dry weather conditions in 2019 and 2020. Significantly higher mortality and crown dieback were observed in trees that showed premature leaf senescence during summer 2018, as well as for trees growing on drier sites, *i.e*. where average CWB was more negative. Similarly, the frequency of bleeding cankers and bark beetles as typical symptoms of secondary damage increased for at least 2 years after the extreme drought in 2018. Partial decreases in crown dieback and bleeding cankers in the third year suggest that European beech can slowly recover, provided there are no additional disturbances or recurring droughts. Our results also reveal that larger and taller trees (defined by DBH and/or tree height) were more affected by drought. Finally, elevated crown dieback was found on soils with higher gravel and clay content, whereas bleeding cankers and bark beetle holes occurred more frequently in trees that had a higher proportion of crown dieback, indicating a predisposition for drought damage.

### Drought‐induced mortality

Our repeated beech monitoring after the 2018 drought showed substantial tree mortality 1 year after the drought and a continued increase in the two subsequent years (Fig. 3). The resulting mean annual mortality rate of 2.1% in early leaf fall trees was 1.6 times higher than the long‐term background annual mortality rate of beech (DBH >5 cm; Etzold *et al*. 2019). The observed increasing beech mortality for at least 3 years confirms earlier observations, indicating that severe droughts and heatwaves can not only cause direct heat‐induced damage (Marchin *et al*. 2022) and immediate tree mortality (Williams *et al*. 2013; Schuldt *et al*. 2020) but can also predispose trees to decline, leading to mortality over several years or even decades after a drought (Peterken & Mountford 1996; Cavin *et al*. 2013). Such lagged mortality after drought may be the result of preferential carbon allocation to rebuild damaged tissue, which could lead to carbon starvation in the long term (Trugman *et al*. 2018; Massonnet *et al*. 2021) or of insect and pathogen attacks that contribute to the decline by negatively affecting tree functioning for several years (Anderegg *et al*. 2015a; Huang *et al*. 2020; Kannenberg *et al*. 2020). The years 2014 to 2018 were the five driest years recorded in Central Europe in the 253‐year period 1766–2018 with respect to soil moisture (Moravec *et al*. 2021). They were followed by recurring droughts in 2019 and 2020 **(**Fig. 2 **)**. This extraordinary series of dry years may have aggravated stress on already weakened trees and accelerated vitality decline (Schuldt *et al*. 2020). In summary, impacts of this prolonged drought, evident in long‐term growth declines in large‐scale tree ring chronologies (Cailleret *et al*. 2017; Kannenberg *et al*. 2019; Vitasse *et al*. 2019), may have contributed to the observed mortality. By combining data from several forest health monitoring networks, George *et al*. (2021) found significantly increased beech mortality in Europe over the last 25 years. Primary weaknesses of such assessments based on national forest monitoring networks are the multi‐year inventory intervals and the timing of assessments that may dilute the climate signal on mortality and thus underestimate mortality rates (Hülsmann *et al*. 2016; Hember *et al*. 2017). In order to more efficiently detect effects of severe drought on tree decline, high spatial and temporal resolution in forest monitoring should be re‐considered (Hartmann *et al*. 2018; Rohner *et al*. 2021). Such fine‐scale assessments were initiated during the forest decline debate of the 1980s, but efforts were reduced again after the year 2000 (Ferretti 2021). Furthermore, combining ground‐based monitoring with remote‐sensing methods is important to bridge spatial scales, as suggested by the IUFRO‐initiated International Tree Mortality Network (Hartmann *et al*. 2018).

Although we identified logging as a cause of additional mortality that we were able to disentangle from drought‐induced mortality (Table S2), the large number of trees lost through logging renders our interpretation of crown dieback and secondary damage conservative, given that the percentage of felled early leaf fall trees was double that of control trees and logged early leaf fall trees showed almost four times larger crown dieback (23.0 ± 0.6% *versus* 6.0 ± 0.4%). Nevertheless, the relatively low average dieback values of the trees before logging suggest that crown dieback increased drastically between the last survey and the logging, or that not only severely damaged or dead trees but also quite healthy trees were cut, probably for operational and technical reasons.

### Premature leaf senescence as a preliminary sign of drought‐induced damage

Drought‐induced leaf discoloration and early leaf fall were widely observed phenomena in summer 2018 (Schuldt *et al*. 2020; Bigler & Vitasse 2021) and remote‐sensing data confirmed that large parts of the natural range of European beech were affected (Baltensweiler *et al*. 2020; Brun *et al*. 2020; Sturm *et al*. 2022). Our ground‐measured data show that trees with early leaf fall in 2018 had increased proportions of crown dieback and tree mortality, as well as an increased susceptibility to secondary damage for at least two consecutive years as compared to trees that exhibited normal senescence timing in 2018 **(**Figs 3 and 4 **)**. These results suggest that early leaf fall is an indicator of a predisposition of beech trees for decline incited by the drought event (Manion 1991; Walthert *et al*. 2021). They are in line with our predictions and with recent findings of Walthert *et al*. (2021), demonstrating clear relationships between soil water potential, leaf water potential and crown dieback in years following an extreme drought. Although early leaf fall may initially have been a physiological response to reduce water loss and xylem tension with the aim of avoiding embolism (Wolfe *et al*. 2016), our results suggest that the 2018 drought was so severe that it was – particularly in dry regions – an inciting event causing widespread hydraulic failure (Brodribb *et al*. 2020; Wohlgemuth *et al*. 2020; Arend *et al*. 2021; Walthert *et al*. 2021; Arend *et al*. 2022) with subsequent crown‐dieback, which ultimately leads to higher tree mortality (Chakraborty *et al*. 2017; Leuschner 2020; Schuldt *et al*. 2020). In these regions, the observed early leaf senescence during summer 2018 was, in fact, an indicator of stress and a predisposition for eventual crown dieback and tree mortality.

The presence of bleeding cankers and bark beetle holes increased for at least 2 years after the drought, with early leaf fall trees being more frequently affected than control trees **(**Fig. 4 **)**, which also suggests that these trees were predisposed for decline. Both secondary damage symptoms were also positively correlated with the percentage of crown dieback, suggesting that drought legacy had increased susceptibility of these beech trees to secondary damage (Schuldt *et al*. 2020). This phenomenon is particularly known for bark beetle attacks in Norway spruce (Biedermann *et al*. 2019) but has also been observed in other species (*e.g*. Bigler *et al*. 2006). For beech, it has been shown that drought‐affected trees are more vulnerable to infestations by the beech splendour beetle (*Agrilus viridis*) and microfungi (Bolte *et al*. 2009; Jung 2009; Gösswein *et al*. 2017; Corcobado *et al*. 2020), acting as factors contributing to decline. *Agrilus* infestations were also observed in our study, but the beetle occurred mainly in the canopy and its population size could thus not be quantified. As contributing factors, such insect infestations can exacerbate the vitality decline of trees, driving the ‘decline spiral’ towards mortality (Manion 1991). Accordingly, Jung (2009) related beech decline in Central Europe after the 2003 drought to the interaction between climatic extremes (inciting factors) and *Phytophthora* spp. infections (contributing factor). Yet, we detected only a few *Phytophthora* spp. infections in our study trees (data not shown), suggesting that the occurrence of bleeding cankers was rather due to physiological stress or other non‐targeted biotic agents.

Contrary to our expectation that premature leaf senescence eventually leads to partial or full crown mortality and to secondary damage, the proportions of crown dieback and the presence of bleeding cankers and bark beetle holes remained constant or slightly decreased between 2020 and 2021 **(**Figs 3 and 4 **)**, indicating partial recovery. The reduced proportion of crown dieback in the rather wet summer of 2021 was likely due to regrowth of new crown biomass, such as twigs and leaves (Jump *et al*. 2017; Gessler *et al*. 2020), as indicated by decreasing crown transparency and an increasing proportion of trees that produced epicormic branches in the crown (Figure S1, Table S3). While full recovery from mild droughts is possible within a short time, more severe events cause structural damage that can only be compensated by the regrowth of new tissue, which is a relatively slow process (Ruehr *et al*. 2019). On the one hand, rapid recovery of radial growth within a few years after drought has repeatedly been described in beech (*e.g*. Bolte *et al*. 2010; Scharnweber *et al*. 2011; Pretzsch *et al*. 2020). On the other hand, longer‐term growth decline (Peterken & Mountford 1996; Cavin *et al*. 2013) and aggravated negative growth impacts by the consecutive summer drought 2019 have also been reported (Schnabel *et al*. 2021). The 3‐year observation period of our study was too short to quantify to what extent drought‐damaged beech trees can recover, because full recovery of tree vigour can take much longer, and delayed mortality may also occur several years to decades post‐drought (Trugman *et al*. 2018), particularly under recurring droughts (Mitchell *et al*. 2016).

### Regional‐ and local‐scale drivers of drought damage

Our monitoring showed that drought damage often affected patches of several beech trees next to unaffected individuals. Such small‐scale variations in damage patterns have been described repeatedly, but the causes are still unclear (Bréda *et al*. 2006; Trugman *et al*. 2021). These studies suggested local variability in site conditions, such as soil properties, microtopography and stand structure, as predisposing factors for the variation in damage on small spatial scales. On a regional scale, our results showed higher tree mortality, higher degrees of crown dieback and more bleeding cankers in the drier regions, as indicated by the negative relation between these responses and the CWB **(**Table 2 **)**. These findings are in line with our hypothesis that the proportions of mortality and secondary damage increase with increasing CWB deficit. Beech trees in drier regions operate closer to their physiological limits, and thus the extreme conditions of the 2018 drought and/or the sequence of multiple drought years in a row pushed them beyond these limits. The fact that regional climate is an important factor predisposing for drought damage was confirmed by evaluations of forest inventory data across the entire species range of beech in Europe, demonstrating that climatic drought intensity was the most important driver for beech mortality (Neumann *et al*. 2017; Archambeau *et al*. 2020). Mortality of beech and several other tree species was also related to soil moisture in an analysis of ICP Forests data over 25 years (George *et al*. 2021) and mortality was related to water stress in several tree species across North America (Hember *et al*. 2017). In contrast, no long‐term trend of increased beech mortality under drier conditions has been detected in data from the Swiss forest health monitoring network across broad ecological amplitudes (Etzold *et al*. 2019).

Contrary to our expectations and in contrast to reports of increased drought‐induced beech mortality on shallow soils (Mueller *et al*. 2005; Allen *et al*. 2010; Schuldt *et al*. 2020), soil depth did not significantly predispose beech for drought damage in our study. One reason might be that sites with very shallow soils were not well represented. Also, the modelled soil parameters (Baltensweiler *et al*. 2021) may not have reproduced small‐scale variation in soil properties with sufficient accuracy. We found higher crown dieback on soils with higher gravel and clay content, which may reduce soil water retention capacity, as high gravel content increases soil drainage whereas high clay content reduces the amount of extractable water (Hillel 1980). This result is in line with reports by Bréda *et al*. (2006) and Crouchet *et al*. (2019), suggesting small‐scale variation in soil parameters as driving factors for the diffuse or patchy distribution of drought‐damaged trees in forest stands. Likewise, Obladen *et al*. (2021) identified soil properties as key drivers of drought‐induced beech mortality in central Germany. Similar to our results, they reported significant growth reduction in beech and Norway spruce at the study site with the highest soil clay content. Recurring droughts may have resulted in soil water depletion, while clay shrinkage might have truncated roots (Sanders *et al*. 2012).

As hypothesized, we found higher mortality, greater proportions of crown dieback and higher occurrence of bleeding cankers in larger trees **(**Table 2 **)**. Elevated mortality in taller trees was reported from natural beech forests in Sweden and Ukraine, whereas no or an opposite size effect was described in Germany and Switzerland (Fuentes *et al*. 2010; Hülsmann *et al*. 2016). Likewise, Hember *et al*. (2017) found decreasing sensitivity to soil water deficit with increasing tree height in an analysis of North American forest inventory data. A possible explanation for these divergent findings may be that the influence of tree size on drought susceptibility depends on the severity of a drought, as larger trees were found to be more resilient to mild droughts but more vulnerable to severe events than smaller trees (Bottero *et al*. 2021). Additional factors, such as forest openings due to management, may potentially have amplified crown damage by increasing sun‐burn of leaves. However, they are unlikely to have affected crown condition at our study sites because the degree of crown damage did not correlate with the proportion of missing trees (stumps) in the neighbourhood (data not shown). Tree mortality in our study increased with increasing competition from neighbouring trees. Similarly, Klesse *et al*. (2022) found that drought‐induced crown damage was more severe in shorter and slower‐growing beech trees that experience stronger competition. These differences among individual trees further emphasize the importance of local‐scale processes in determining drought effects on trees.

## CONCLUSIONS

Our large‐scale beech monitoring for three consecutive years following the extreme 2018 hot drought found increasing crown and tree mortality, as well as secondary damage symptoms after early leaf senescence, thus providing evidence for adverse drought legacy effects. These findings suggest that premature leaf senescence was an indicator of predisposition of beech trees for decline on dry sites. The severe drought in summer 2018 predisposed trees to a decline, which may end in tree mortality, particularly if contributing insect and pathogen attacks exacerbate the tree damage. Recurring droughts in the two subsequent years have likely aggravated the stress for beech trees. While climatic drought intensity determined drought damage at regional scale, soil properties, tree and stand characteristics modulated local damage patterns, resulting in patches of trees suffering severe damage located in proximity to patches of more drought‐resistant trees. Crown tissue regrowth in later years of the monitoring indicates the beginning of partial canopy recovery, which may ultimately result in either long‐term tree resilience or increased drought susceptibility. Under future climate change with increasing frequency and intensity of droughts and heat spells, further beech decline may be expected on dry sites in northern Switzerland. This underlines the importance of adapting forest management to the changing climate, for example by promoting mixed stands with better heat‐ and drought‐adapted species in order to increase forest resilience.

## Supporting information


**Table S1.** Mean temperature, precipitation sum and climatic water balance (CWB) for the growing season (April to September) in the three regions Basel (BL), Schaffhausen (SH), and Zurich (ZH) for the years 2018 to 2021 and the climate norm period 1981–2010.
**Table S2.** Cumulative percentage of dead and logged trees averaged per tree group (mean ± 1 SE) of early leaf fall and control trees in the three regions Basel (BL), Schaffhausen (SH) and Zurich (ZH) from 2018 to 2021.
**Table S3.** Proportion of crown dieback (mean ± 1 SE) (A), presence of bleeding cankers (B), presence of bark beetle holes (C) and crown transparency (D) of early leaf fall and control trees in the three regions Basel (BL), Schaffhausen (SH) and Zurich (ZH).
**Table S4.** Average values of explanatory variables (mean ± 1 SE) of early leaf fall and control trees in the three regions Basel (BL), Schaffhausen (SH) and Zurich (ZH).
**Figure S1.** Development of crown transparency (mean ± SE) in early leaf fall (dashed lines) and control trees (solid lines) in the three regions Basel (BL), Schaffhausen (SH) and Zurich (ZH) in northern Switzerland from 2019 to 2021. Only trees were included that had observations in all surveys (N = 745; for numbers see also Table S3).
