# Supplementary material for: European beech dieback after premature leaf senescence during the 2018 drought in northern Switzerland
Source: Plant Biol (Stuttg). 2022 Oct 18;24(7):1132–45. doi: 10.1111/plb.13467 (PMC10092601; doi:10.1111/plb.13467)
Supplement: Supplementary file 1 — Table S1. Mean temperature, precipitation sum and climatic water balance (CWB) for the growing season (April to September) in the three regions Basel (BL), Schaffhausen (SH), and Zurich (ZH) for the years 2018 to 2021 and the climate norm period 1981–2010. Table S2. Cumulative percentage of dead and logged trees averaged per tree group (mean ± 1 SE) of early leaf fall and control trees in the three regions Basel (BL), Schaffhausen (SH) and Zurich (ZH) from 2018 to 2021. Table S3. Proportion of crown dieback (mean ± 1 SE) (A), presence of bleeding cankers (B), presence of bark beetle holes (C) and crown transparency (D) of early leaf fall and control trees in the three regions Basel (BL), Schaffhausen (SH) and Zurich (ZH). Table S4. Average values of explanatory variables (mean ± 1 SE) of early leaf fall and control trees in the three regions Basel (BL), Schaffhausen (SH) and Zurich (ZH). Figure S1. Development of crown transparency (mean ± SE) in early leaf fall (dashed lines) and control trees (solid lines) in the three regions Basel (BL), Schaffhausen (SH) and Zurich (ZH) in northern Switzerland from 2019 to 2021. Only trees were included that had observations in all surveys (N = 745; for numbers see also Table S3). [file PLB-24-1132-s001.docx]

*Plant Biology Special Issue «Responses of European forests to global change-type droughts»*

European beech dieback after premature leaf senescence during the 2018 drought in northern Switzerland

**Running Title:** Drought effects on European beech

Esther R. Frei^1,2,3,4,*^, Martin M. Gossner^1,3,5^, Yann Vitasse^1,3^, Valentin Queloz^1,3^, Vivanne Dubach^1^, Arthur Gessler^1,3,5^, Christian Ginzler^1,3^, Frank Hagedorn^1,3^, Katrin Meusburger^1,3^, Maurice Moor^1^, Eva Samblás Vives^1,6^, Andreas Rigling^1,3,5^, Ide Uitentuis^1^, Georg von Arx^1,3,7^, Thomas Wohlgemuth^1,3^

^1^Swiss Federal Institute for Forest, Snow and Landscape Research WSL, Zuercherstrasse 111, 8903 Birmensdorf, Switzerland

^2^WSL Institute for Snow and Avalanche Research SLF, 7260 Davos Dorf, Switzerland

^3^SwissForestLab, 8903 Birmensdorf, Switzerland

^4^Climate Change and Extremes in Alpine Regions Research Centre CERC, 7260 Davos Dorf, Switzerland

^5^Department of Environmental Systems Science, ETH Zurich, 8092 Zurich, Switzerland

^6^Autonomous University of Barcelona (UAB), Cerdanyola del Valles, Spain

^7^Oeschger Centre for Climate Change Research, University of Bern, 3012 Bern, Switzerland

*Correspondence: Esther R. Frei, esther.frei@wsl.ch

**Keywords:** bark beetles; bleeding cankers; climatic water balance; crown dieback; *Fagus sylvatica*; tree mortality

Supporting Information

**Table S1.** Mean temperature, precipitation sum and climatic water balance (CWB) for the growing season (April to September) in the three regions Basel (BL), Schaffhausen (SH), and Zurich (ZH) for the years 2018 to 2021 and the climate norm period 1981-2010.

|  | Temperature [°C] | | |  | Precipitation [mm] | | |  | CWB [mm] | | |
| --- | --- | --- | --- | --- | --- | --- | --- | --- | --- | --- | --- |
|  | BL | SH | ZH |  | BL | SH | ZH |  | BL | SH | ZH |
| 2018 | 18.6 | 18.0 | 17.7 |  | 324 | 414 | 434 |  | -374 | -257 | -241 |
| 2019 | 16.7 | 16.2 | 16.0 |  | 480 | 514 | 558 |  | -163 | -86 | -45 |
| 2020 | 17.6 | 16.6 | 16.5 |  | 349 | 392 | 462 |  | -322 | -233 | -175 |
| 2021 | 15.8 | 15.0 | 14.9 |  | 578 | 688 | 676 |  | 7 | 144 | 115 |
| 1981-2010 | 15.9 | 15.2 | 15.0 |  | 498 | 534 | 618 |  | -51 | -51 | +66 |

Regional climate data for each site was derived from the nearest weather station: BL: Basel / Binningen, 318 m. ü. M., 47.54°N / 7.58 °E, SH: Schaffhausen, 438 m. ü. M., 47.68 °N / 8.62 °E, ZH: Zürich / Affoltern, 444 m. ü. M., 47.42 °N/ 8.51 °E. ©MeteoSwiss.

**Table S2.** Cumulative percentage of dead and logged trees averaged per tree group (mean ± 1 SE) of early leaf fall and control trees in the three regions Basel (BL), Schaffhausen (SH) and Zurich (ZH) from 2018 to 2021.

|  | No | Aug  2018 |  | Apr  2019 |  | Aug  2019 |  | May  2020 |  | Aug  2020 |  | Aug  2021 |  |
| --- | --- | --- | --- | --- | --- | --- | --- | --- | --- | --- | --- | --- | --- |
|  |  | dead | logged | dead | logged | dead | logged | dead | logged | dead | logged | dead | logged |
| BL Early | 303 | 0.0±0.0 | 0.0±0.0 | 1.0±0.6 | 6.6±2.7 | 3.3±1.0 | 11.8±3.8 | 3.9±1.0 | 32.2±5.1 | 5.6±1.2 | 32.5±5.2 | 7.2±1.6 | 39.8±5.4 |
| BL Control | 92 | 0.0±0.0 | 0.0±0.0 | 0.0±0.0 | 0.0±0.0 | 1.1±1.1 | 0.0±0.0 | 1.1±1.1 | 11.6±6.7 | 1.1±1.1 | 11.6±6.7 | 2.1±1.4 | 11.6±6.7 |
|  |  |  |  |  |  |  |  |  |  |  |  |  |  |
| SH Early | 300 | 0.0±0.0 | 0.0±0.0 | 2.3±0.8 | 1.7±1.1 | 4.7±1.2 | 1.7±1.1 | 5.3±1.3 | 5.3±2.2 | 6.3±1.5 | 5.7±2.3 | 10.3±2.0 | 16.0±4.1 |
|  |  |  |  |  |  |  |  |  |  |  |  |  |  |
| ZH Early | 221 | 0.0±0.0 | 0.0±0.0 | 0.0±0.0 | 2.9±1.9 | 3.0±2.0 | 2.5±1.8 | 3.0±2.0 | 3.6±2.0 | 3.4±2.0 | 3.9±2.1 | 3.8±2.0 | 10.4±3.4 |
| ZH Control | 47 | 0.0±0.0 | 0.0±0.0 | 0.0±0.0 | 9.1±9.1 | 0.0±0.0 | 9.1±9.1 | 0.0±0.0 | 9.1±9.1 | 0.0±0.0 | 16.4±11.1 | 0.0±0.0 | 18.2±10.9 |
|  |  |  |  |  |  |  |  |  |  |  |  |  |  |
| ALL Early | 824 | 0.0±0.0 | 0.0±0.0 | 1.1±0.3 | 3.7±1.2 | 3.7±0.8 | 5.4±1.5 | 4.1±0.9 | 14.0±2.2 | 5.1±0.9 | 14.4±2.3 | 7.2±1.1 | 22.4±2.7 |
| ALL Control | 139 | 0.0±0.0 | 0.0±0.0 | 0.0±0.0 | 3.3±3.3 | 0.7±0.7 | 3.3±3.3 | 0.7±0.7 | 10.7±5.3 | 0.7±0.7 | 13.3±5.8 | 1.3±0.9 | 14.0±5.8 |
|  |  |  |  |  |  |  |  |  |  |  |  |  |  |
| **All** | **963** | **0.0±0.0** | **0.0±0.0** | **0.7±0.5** | **3.7±1.1** | **2.1±0.8** | **5.1±1.4** | **2.3±1.0** | **13.6±2.1** | **3.0±1.3** | **14.2±2.1** | **4.4±1.9** | **21.2±2.5** |

No, initial number of trees in August 2018. Dead, Logged, cumulative percentages of dead and logged trees with respect to the initial numbers of trees in this group.

**Table S3.** Proportion of crown dieback (mean ± 1 SE) (A), presence of bleeding cankers (B), presence of bark beetle holes (C) and crown transparency (D) of early leaf fall and control trees in the three regions Basel (BL), Schaffhausen (SH) and Zurich (ZH).

|  |  | No | Aug 2018 | Apr 2019 | Aug 2019 | Aug 2020 | Aug 2021 |
| --- | --- | --- | --- | --- | --- | --- | --- |
| (A) Crown dieback | |  |  |  |  |  |  |
|  | BL Early | 178 | 0.2 ± 0.1 | 13.1 ± 1.4 | 29.3 ± 2 | 35.4 ± 2.6 | 23.4 ± 2.3 |
|  | BL Control | 81 | 0.0 ± 0.0 | 5.2 ± 0.4 | 9.4 ± 0.8 | 5.1 ± 1.3 | 5.3 ± 1.4 |
|  |  |  |  |  |  |  |  |
|  | SH Early | 249 | 1.8 ± 0.3 | 16.1 ± 1.4 | 27.2 ± 1.8 | 30.2 ± 2.2 | 20.7 ± 2.0 |
|  |  |  |  |  |  |  |  |
|  | ZH Early | 196 | 1.2 ± 0.2 | 8.0 ± 0.8 | 14.8 ± 1.2 | 22.3 ± 2.1 | 10.4 ± 1.4 |
|  | ZH Control | 41 | 0.2 ± 0.2 | 3.2 ± 0.5 | 5.5 ± 0.6 | 3.7 ± 1 | 1.3 ± 0.4 |
|  |  |  |  |  |  |  |  |
|  | All Early | 623 | 1.2 ± 0.1 | 12.7 ± 0.7 | 23.9 ± 1 | 29.2 ± 1.3 | 18.2 ± 1.1 |
|  | All Control | 122 | 0.1 ± 0.1 | 4.5 ± 0.4 | 8.1 ± 0.6 | 4.6 ± 0.9 | 4.0 ± 1.0 |
|  |  |  |  |  |  |  |  |
|  | **All trees** | **745** | **1.0 ± 0.1** | **11.3 ± 0.6** | **21.3 ± 0.9** | **25.3 ± 1.2** | **15.9 ± 1.0** |
| (B) Bleeding cankers | |  |  |  |  |  |  |
|  | BL Early | 178 | 1.7 | 14.1 | 22.5 | 23.2 | 5.8 |
|  | BL Control | 81 | 0.0 | 6.2 | 12.3 | 16.0 | 2.5 |
|  |  |  |  |  |  |  |  |
|  | SH Early | 249 | 0.4 | 16.9 | 24.5 | 26.3 | 3.2 |
|  |  |  |  |  |  |  |  |
|  | ZH Early | 196 | 1.5 | 16.9 | 26.5 | 10.2 | 4.6 |
|  | ZH Control | 41 | 0.0 | 4.9 | 4.9 | 0.0 | 2.4 |
|  |  |  |  |  |  |  |  |
|  | All Early | 623 | 1.1 | 16.1 | 24.6 | 20.1 | 4.4 |
|  | All Control | 122 | 0.0 | 5.7 | 9.8 | 10.7 | 2.5 |
|  |  |  |  |  |  |  |  |
|  | **All trees** | **745** | **0.9** | **14.4** | **22.1** | **18.5** | **4.1** |
| (C) Bark beetles | |  |  |  |  |  |  |
|  | BL Early | 178 | - | 0.0 | 6.2 | 23.6 | 31.5 |
|  | BL Control | 81 | - | 1.2 | 3.7 | 14.8 | 16.0 |
|  |  |  |  |  |  |  |  |
|  | SH Early | 249 | - | 1.2 | 12.9 | 18.1 | 20.1 |
|  |  |  |  |  |  |  |  |
|  | ZH Early | 196 | - | 2.6 | 6.6 | 10.7 | 18.4 |
|  | ZH Control | 41 | - | 2.4 | 4.9 | 9.8 | 12.2 |
|  |  |  |  |  |  |  |  |
|  | All Early | 623 | - | 1.3 | 9.0 | 17.3 | 22.8 |
|  | All Control | 122 | - | 1.6 | 4.1 | 13.1 | 14.8 |
|  |  |  |  |  |  |  |  |
|  | **All trees** | **745** | **-** | **1.3** | **8.2** | **16.6** | **21.5** |
| (D) Crown transparency | |  |  |  |  |  |  |
|  | BL Early | 178 | 43.2 ± 1.3 | - | - | 52.9 ± 1.7 | 34.4 ± 1.7 |
|  | BL Control | 81 | 28.5 ± 1.2 | - | - | 32.4 ± 1.7 | 25.6 ± 1.3 |
|  |  |  |  |  |  |  |  |
|  | SH Early | 249 | 44.4 ± 0.9 | - | - | 45.0 ± 1.6 | 30.1 ± 1.7 |
|  |  |  |  |  |  |  |  |
|  | ZH Early | 196 | 39.3 ± 1.0 | - | - | 39.2 ± 1.5 | 26.2 ± 1.1 |
|  | ZH Control | 41 | 27.3 ± 1.2 | - | - | 15.1 ± 2.1 | 18.3 ± 1.2 |
|  |  |  |  |  |  |  |  |
|  | All Early | 623 | 42.4 ± 0.6 | - | - | 45.4 ± 1.0 | 30.1 ± 0.9 |
|  | All Control | 122 | 28.1 ± 0.9 | - | - | 26.3 ± 1.5 | 23.1 ± 1.0 |
|  |  |  |  |  |  |  |  |
|  | **All trees** | **745** | **39.9 ± 0.6** | **-** | **-** | **42.4 ± 0.9** | **29.0 ± 0.8** |

No, number of analyzed trees. ‘-‘, no assessments for the respective parameter were made (bark beetle holes in summer 2018, crown transparency in 2019).

**Table S4.** Average values of explanatory variables (mean ± 1 SE) of early leaf fall and control trees in the three regions Basel (BL), Schaffhausen (SH) and Zurich (ZH).

|  | No | Clay content [wt-%] | Competition index  [m^-1^] | CWB  [mm] | DBH [cm] | Forest edge distance [m] | Gravel content [vol-%] | Soil depth [cm] | Soil pH | Tree height [m] |
| --- | --- | --- | --- | --- | --- | --- | --- | --- | --- | --- |
| BL Early | 178 | 26±0.6 | 1.1±0.1 | -138±4 | 44.8±0.9 | 78.5±6.0 | 36±0.8 | 74±1.8 | 5.4±0.1 | 26.9±0.3 |
| BL Control | 81 | 28±0.8 | 1.2±0.1 | -124±6 | 45.6±1.4 | 56.8±5.7 | 36±1.0 | 61±2.7 | 5.9±0.1 | 29.8±0.5 |
|  |  |  |  |  |  |  |  |  |  |  |
| SH Early | 249 | 27±0.2 | 1.2±0.0 | -202±1 | 40.8±0.7 | 90.2±5.3 | 21±0.3 | 63±1.7 | 5.1±0.1 | 27.8±0.2 |
|  |  |  |  |  |  |  |  |  |  |  |
| ZH Early | 196 | 21±0.2 | 1.1±0.1 | -6±4 | 51.0±1.1 | 25.3±2.1 | 18±0.2 | 69±1.6 | 5.4±0.1 | 29.5±0.2 |
| ZH Control | 41 | 22±0.3 | 0.9±0.1 | 7±7 | 49.9±2.0 | 20.1±2.0 | 18±0.6 | 67±2.6 | 5.2±0.2 | 32.2±0.5 |
|  |  |  |  |  |  |  |  |  |  |  |
| All Early | 623 | 25±0.2 | 1.1±0.0 | -122±4 | 45.2±0.5 | 66.4±3.0 | 24±0.4 | 68±1 | 5.3±0.1 | 28.0±0.1 |
| All Control | 122 | 26±0.6 | 1.1±0.1 | -80±7 | 47.0±1.1 | 44.5±4.1 | 30±1.0 | 63±2.0 | 5.7±0.1 | 30.6±0.4 |
|  |  |  |  |  |  |  |  |  |  |  |
| **All trees** | **745** | **25±0.2** | **1.1±0.0** | **-115±3** | **45.5±0.5** | **62.8±2.6** | **25±0.4** | **67±0.9** | **5.3±0.0** | **28.5±0.1** |

No, number of analyzed trees. For variable descriptions see Table 1.


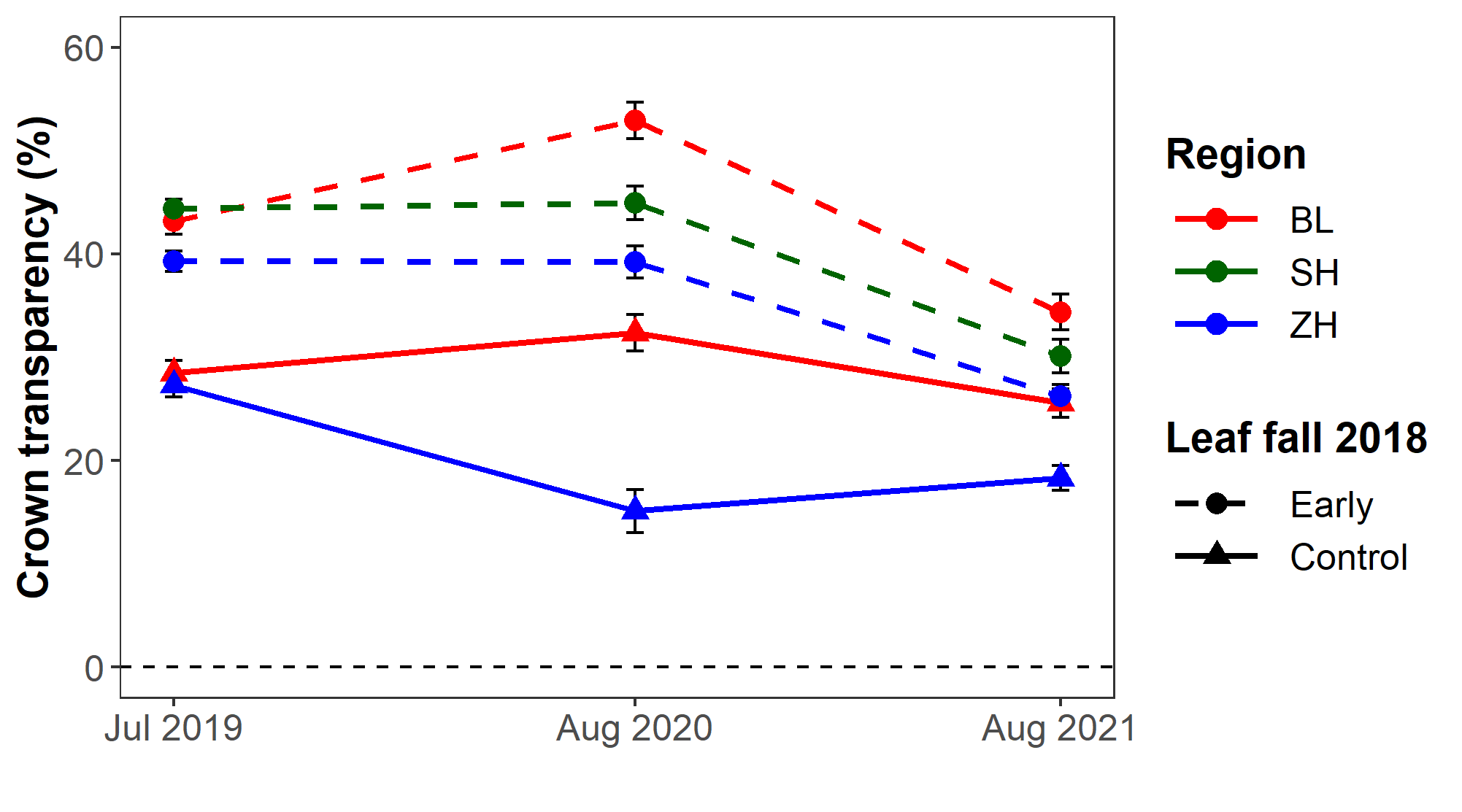


**Figure S1.** Development of crown transparency (mean ± SE) in early leaf fall (dashed lines) and control trees (solid lines) in the three regions Basel (BL), Schaffhausen (SH) and Zurich (ZH) in northern Switzerland from 2019 to 2021. Only trees were included that had observations in all surveys (N = 745; for numbers see also Supplementary Table S3).
